# The effect of the interaction between aquaporin 0 (AQP0) and the filensin tail region on AQP0 water permeability

**Published:** 2011-12-13

**Authors:** Yosuke Nakazawa, Mikako Oka, Katsuya Furuki, Akiko Mitsuishi, Emi Nakashima, Makoto Takehana

**Affiliations:** 1 Department of Molecular Function and Physiology, Faculty of Pharmacy, Keio University, Tokyo, Japan; 2 Department of Parmaceutics, Faculty of Pharmacy, Keio University, Tokyo, Japan

## Abstract

**Purpose:**

To study the interaction between the lens-specific water channel protein, aquaporin 0 (AQP0) and the lens-specific intermediate filament protein, filensin, and the effect of this interaction on the water permeability of AQP0. The effect of other factors on the interaction was also investigated.

**Methods:**

Expression plasmids were constructed in which glutathione-S-transferase (GST) was fused to the AQP0 COOH-terminal region (GST-AQP0-C), which contains the major phosphorylation sites of the protein. Plasmids for AQP0 COOH-terminal mutants were also constructed in which one, three or five sites were pseudophosphorylated, and the proteins expressed from these GST-fusion plasmids were assayed for their interaction with lens proteins. Expressed recombinant GST-fusion proteins were purified using glutathione beads and incubated with rat lens extract. Western blotting was used to identify the lens proteins that interacted with the GST-fusion proteins. Filensin tail and rod domains were also expressed as GST-fusion proteins and their interactions with AQPO were analyzed. Additionally, the water permeability of AQP0 was calculated by expressing AQP0 with or without the filensin peptide on the cell membrane of *Xenopus* oocytes by injecting cRNAs for AQP0 and filensin.

**Results:**

The GST-AQP0-C construct interacted with the tail region of lens filensin and the GST-filensin-tail construct interacted with lens AQP0, but the GST-filensin-rod construct did not interact with AQP0. GST-AQP0-C also interacted with a purified recombinant filensin-tail peptide after cleavage from GST. The AQP0/filensin-tail interaction was not affected by pseudophosphorylation of the AQP0 COOH-terminal tail, nor was it affected by changes in pH. *Xenopus* oocytes expressing AQP0 on the plasma membrane showed increased water permeability, which was lowered when the filensin COOH-terminal peptide cRNA was coinjected with the cRNA for AQP0.

**Conclusions:**

The filensin COOH-terminal tail region interacted with the AQP0 COOH-terminal region and the results strongly suggested that the interaction was direct. It appears that interactions between AQP0 and filensin helps to regulate the water permeability of AQP0 and to organize the structure of lens fiber cells, and may also help to maintain the transparency of the lens.

## Introduction

Aquaporins are a family of ubiquitous membrane proteins that form channels allowing the permeation of water and small, neutral molecules, such as glycerol, across cell membranes [1,2]. Aquaporin 0 (AQP0), also known as major intrinsic peptide (MIP) 26, is the most abundant fiber cell membrane protein in lens. AQP0 is also expressed in retinal amacrine cells, retinal ganglion cells and liver cells [3,4]. AQP0 constitutes more than 60% of the total membrane protein content of fiber cells [5,6] and consists of six trans-membrane helices with both the NH_2_-and COOH-termini localized to the cell cytoplasm. AQP0 exists as a tetramer and each subunit contains an individual aqueous pore [7]. Compared with other aquaporins, AQP0 has special properties, including a very limited ability to transport water [2,8]. There may be a specific reason for the low permeability of this lens water channel but it is not yet known.

However, it is known that the permeability of AQP0 is regulated by many factors; for example, acidic pH increases the water permeability of bovine AQP0 [9] and low levels of calcium ions and calmodulin inhibitors also increase AQP0 permeability [9]. Calmodulin binds to AQPO at the COOH-terminal region and it was reported that the phosphorylation of AQP0 COOH-terminal peptides lowers its affinity for calmodulin [10], which suggests that AQP0 COOH-terminal phosphorylation regulates water permeability. Zinc also increases the water permeability of AQP0 [11], while truncation of the AQP0 COOH-terminal tail results in the loss of its transporter ability [2].

The COOH-terminal region of AQP0 contains several phosphorylation sites. There are six serines in the COOH-terminal tail and it is thought that five of the six serines (serine 229, 231, 234, 243, and 245) are phosphorylated [10,12-14]. As mentioned above, the phosphorylation of serine residues affects the interaction between calmodulin and AQP0 [10]. In addition to phosphorylation, many other factors affect AQP0 permeability and, in this study, we have investigated the possibility that filensin, a lens-specific intermediate filament protein, also plays a role in controlling the water permeability of AQP0.

Beaded filaments are lens fiber cell-specific intermediate filaments [15,16] composed of two proteins, filensin [17,18] and phakinin [19,20]. Similar to other intermediate filament proteins, the structure of filensin consists of a head domain, a rod domain that can be divided into three subdomains (1A, 1B, and 2), and a COOH-terminal tail domain. Filensin is a 94 kDa intermediate filament protein, which is processed into two smaller molecular weight proteins of 50 and 38 kDa in the normal lens [21,22]. Both these smaller filensin fragments contain the rod region and have been localized to the central region of lens fiber cells in the deep cortex of the lens. In contrast, the tail region of filensin is localized to sub-cellular membrane regions of lens fiber cells [22], probably because the COOH-terminal fragment of filensin is myristoylated and has strong membrane-binding properties [23]. Previous studies have shown that the deletion of filensin or phakinin expression in mice by gene targeting causes cataracts and that some forms of hereditary cataracts in humans are caused by filensin or phakinin mutations [24-28], which suggests that beaded filaments play an important role in lens transparency. From these reports, the rod and tail domains of filensin appear to have different roles in lens fiber cells, and it is possible that each region of the filensin protein may have a particular role to play in the maintenance of lens transparency. The COOH-terminal region of AQP0 is known to interact with filensin [29,30] but the filensin domain with which it interacts is not yet known.

In this study, expression vectors were constructed for the APQ0 COOH-terminal peptide and for pseudophosphorylated AQP0 COOH-terminal peptides to study the interaction between the AQP0 COOH-terminal peptide and filensin. In addition, the effect of the interaction between AQP0 and filensin on water permeability was also investigated.

## Methods

### Animals

Six-week-old Wistar rats and Japanese white rabbits were purchased from Sankyo Labo Service Corporation (Tokyo, Japan). *Xenopus laevis* were purchased from Saitama Experimental Animals Supply Co., Ltd. (Saitama, Japan) The Keio University Animal Research Committee approved all the animal procedures used in the current study.

### Antibodies

The anti-filensin rod domain antibody (raised against the filensin amino acid sequence 133–337) and anti-filensin outer tail domain antibody (raised against the filensin amino acid sequence 510–664) were obtained by immunizing rabbits as described previously [22] and the anti-AQP0 COOH-terminal peptide antibody was raised as described [31]. Briefly, a synthetic peptide corresponding to the COOH-terminal amino acids at positions 253 to 263 (GEPVELKTQAK) in bovine AQP0 was coupled to keyhole limpet hemocyanin (Sigma, St. Louis, MO) and emulsified with Freund’s complete adjuvant (ICN Pharmaceuticals, Mesa, CA). Antiserum against AQP0 was obtained by injecting the adjuvant into Japanese white rabbits.

### Construction of GST-fusion protein expression vectors

Total rat lens RNA was extracted according to Oka et al. [32]. Briefly, lenses were homogenized in TRIZOL® Reagent (Invitrogen Corp., Carlsbad, CA) with a glass-teflon homogenizer. The sample was incubated for 5 min at room temperature before adding chloroform. After centrifugation, the upper aqueous phase was collected, the RNA was precipitated by mixing with isopropyl alcohol, and the RNA pellet was washed with 75% ethanol. The reverse transcriptase polymerase chain reaction (RT–PCR) was performed using the TaKaRa RNA PCR™ Kit (AMV) v3.0 (TaKaRa, Otsu, Japan). The oligonucleotide primer sets used in this study are shown in Table 1. Amplified PCR products from the AQP0 COOH-terminal domain were inserted into the pGEX-6P-1 and/or pGe × −5x-1 expression vectors (GE Healthcare Life Sciences, Buckinghamshire, UK) and transformed into *Escherichia coli* according to the manufacturer's instructions. The method for the construction of the GST-fusion filensin rod domain (GST-filensin-rod) and filensin outer tail domain (GST-filensin-tail) constructs has been described previously [22].

**Table 1:** Primer sets for RT–PCR.

**Gene**	**Forward (5′→3′)**	**Reverse (5′→3′)**
AQP0 COOH- terminal domain	GAATTCCTGCTCTACGACTTTCTCC	GTCGACCGCACCCACATTC
pGEX-a plasmid primer	CCCATCCTGACTTCATGTTG	CCGAAAAGTGCCACCTGACGTC
S235E	GAGACTGGAGATCCTCAAGGGAGCCAG	CTTGAGGATCTCCAGTCTCTCAGAAAC
S229/231E	GCTCAAGGAGGTTGAGGAGAGACTGGAGA	GTCTCTCCTCAACCTCCTTGAGCCGGGGG
S243/245E	CAGACCCGAGGACGAGAATGGACAGCCAG	GTCCATTCTCGTCCTCGGGTCTGGCTCC

### Site-directed mutagenesis

Three pseudophosphorylated forms of the AQP0 COOH-terminal region were constructed using the GST-fusion AQP0 COOH-terminal peptide (GST-AQP0-C) expression plasmid. The primers for the three mutations are shown in Table 1 and the positions for each of the mutations in the AQP0 COOH-terminal peptide are shown in Table 2. For the first mutant plasmid, PCR was performed using the forward mutation primer and reverse pGEX-5x-a plasmid primer set with the GST-AQP0-C construct as a template. A second PCR was performed using the forward pGEX-5x-a plasmid primer and reverse mutation primer set with the same template. The two PCR products were purified and used as the templates for the next PCR with the forward and reverse pGEX-5x-a plasmid primers to amplify the mutated AQP0. The pGEX-5x-a plasmid and amplified mutated AQP0 were digested with EcoR1 and Sal1 restriction enzymes and the resulting restriction fragments were ligated with DNA ligase (TaKaRa). In this study, three site-directed mutants, S235E (1S/E-AQP0), S235E/S229E/S231E (3S/E-AQP0), and S235E/S229E/S231E/S243E/S245E (5S/E-AQP0), were constructed.

**Table 2:** Position of the mutation in AQP0 COOH-terminal peptide.

AQP0 COOH-terminal peptide	217-SLLYDFLLFPRLKSVSERLSILKGARPSDSNGQPEGTGEPVELKTQALK-264
1 site mutated AQP0	217-SLLYDFLLFPRLKSVSERL**E**ILKGARPSDSNGQPEGTGEPVELKTQALK-264
3 site mutated AQP0	217-SLLYDFLLFPRLK**E**V**E**ERL**E**ILKGARPSDSNGQPEGTGEPVELKTQALK-264
5 site mutated AQP0	217-SLLYDFLLFPRLK**E**V**E**ERL**E**ILKGARP**E**D**E**NGQPEGTGEPVELKTQALK-264

### Protein expression and purification

All recombinant GST-fusion proteins were expressed in *E. coli* according to the manufacturer’s instructions. The expressed proteins were collected by centrifugation and re-suspended in phosphate buffered saline (PBS). The cells were sonicated to release intracellular proteins and the extract was then centrifuged at 13,000× g for 20 min to remove cell debris. For the filensin tail domain, the supernatant was collected and filtered through a 0.22 µm pore filter (Millipore Corporate Billerica, MA). The AQP0 peptide and its mutated products formed inclusion bodies when expressed in *E. coli*, and the cell debris participant was added to PBS containing 8 M Urea for 20 min, after which the samples were centrifuged at 13,000× g for 20 min. The supernatant was diluted with five volumes of PBS and was mixed with 1 ml of glutathione beads (GE Healthcare Life Sciences) equilibrated in PBS for 1 h. The glutathione beads with the *E. coli* crude extract were then centrifuged at 800× g for 3 min, the supernatant was eliminated, and the beads were then washed three times with PBS.

### Preparation of rat lens protein extract

Lenses of six-week-old rats were homogenized in homogenizing buffer (25% glycerol, 2 mM EGTA, 25 mM MES, pH 6.8). The homogenate was centrifuged at 12,000× g at 4 °C for 20 min, and then the pellet was washed twice with the homogenizing buffer. For filensin extraction, the pellet was extracted with 8 M urea, 5 mM Tris-HCl, pH 8.0 for 20 min, and the supernatant was collected after centrifugation at 13,000× g for 20 min at room temperature and dialyzed against homogenizing buffer. For AQP0 extraction, the pellet of a lens homogenate was extracted with 5% Triton X-100 in homogenizing buffer, and the supernatant was collected after centrifugation at 13,000× g for 20 min at 4 °C.

### Pull down assay for interaction analysis

The GST-fusion proteins immobilized on glutathione beads were mixed with lens extract or purified recombinant filensin tail peptide and incubated for 15 h at 4 °C. The beads with GST-fusion protein and the interacting protein were washed five times with homogenizing buffer and the loaded washed beads were extracted by adding 40 mM glutathione in homogenizing buffer.

### SDS–PAGE and western blotting analysis

SDS–PAGE was performed using a 12.5% polyacrylamide resolving gel. The resolved proteins were then transferred onto a nitrocellulose membrane, which was blocked with 10% (w/v) skim milk (Wako Pure Chemical Industries Ltd., Osaka, Japan) in TBS (0.9% NaCl, 100 mM Tris-HCl, pH 7.5). Membranes were incubated with antibodies followed by horseradish peroxidase-conjugated anti-rabbit IgG (H^+^L) antibody or anti-mouse IgG (H^+^L) antibody (Bio-Rad Laboratories, Hercules, CA).

Immunoreactive proteins were visualized with 0.05% diaminobenzidine (Wako Pure Chemical Industries Ltd.) and 0.006% hydrogen peroxide or using the ECL Advance Western Blotting Detection Kit (GE Healthcare Life Sciences).

### Expression of AQP0 in *Xenopus* oocytes

The expression of AQP0 on *Xenopus* oocyte membranes was performed according to Németh-Cahalan and Hall [9]. Briefly, adult female *X. laevis* were anesthetized, and stage V and VI oocytes removed and prepared as previously described [8]. The day after isolation, oocytes were injected with 12.5 ng of AQP0 and 12.5 ng of filensin-tail cRNA and maintained in ND96 buffer (96 mM NaCl, 2 mM KCl, 1 mM MgCl2, 1.8 mM CaCl2, 5 mM HEPES, pH 7.5, 2.5 mM Na-pyruvate) supplemented with 100 U/ml penicillin and 100 µg/ml streptomycin at 18 °C.

### Measurement of AQP0 permeability

*Xenopus* oocytes injected with cRNA were transferred to 30% ND96 solution. After transfer to the hypotonic solution, the oocytes were photographed and the volume of each oocyte was calculated by measuring its diameter. The solute permeability coefficient, Pf (µm/s), was calculated using the following equation: Pf=[(1000 × V0)/(S × Vw ×Δosm)]) × (d(Vt/V0)/dt), where Δosm=(solout -solin)=130 mOsm (osmotic solute gradient), V0=9·10–4 cm^3^ (initial oocyte volume), d(V/V0)/dt (relative volume increase in s-1), S=0.045 cm^2^ (initial oocyte surface area), Vw=18 ml/mol [33].

## Results

### Interaction between recombinant AQP0 and beaded filament proteins in lens extract

As described in the Methods section, the recombinant GST-AQP0-C construct was expressed in *E. coli* and purified using glutathione beads. After purification, the beads were incubated with lens extract and the lens proteins that interacted with the AQP0 COOH-terminal peptide were analyzed by western blotting.

**Figure 1:**
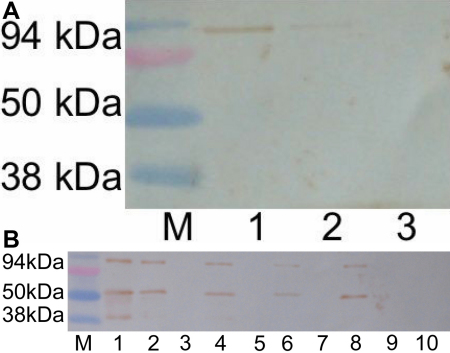
Interaction between GST-AQP0-C and filensin in rat lens extract. **A**: The interaction between purified GST-AQP0-C and lens filensin. Western blotting analysis was performed with anti-filensin outer tail domain antibody. Lane 1: urea fraction of rat lens extract showing 94 kDa filensin. Purified GST-AQP0-C incubated with lens extract (lane 2). Filensin (94 kDa) was detected by anti anti-filensin outer tail domain antibody that interacts with the C-terminal domain of AQP0. Lane 3 contained purified GST-AQP0-C as a control. **B**: The interaction between lens filensin and purified GST-AQP0-C or its pseudophosphorylated forms (1, 3, or 5 sites mutated in the COOH-terminal peptide) was investigated by western blotting with anti-filensin rod antibody. Lane 1: urea fraction of rat lens extract showing 94, 50, and 38 kDa filensin protein bands. Purified GST-AQP0-C or GST-AQP0-C with 1, 3, or 5 pseudophosphorylated sites interacts with the 94 and 50 kDa filensin bands but not with the 38 kDa band (lanes 2, 4, 6, and 8, respectively). In the absence of lens extract, neither the purified GST-AQP0-C nor the 1, 3, or 5 site-mutated AQP0-C constructs interacted with the anti-filensin rod antibody (lanes 3, 5, 7, and 9, respectively). As a further control, when GST alone was incubated with lens extract, it did not interact with lens filensin (lane 10). Lane M in **A** and **B** shows the molecular markers.

The anti-filensin outer tail domain antibody was used to investigate the interaction between lens extract proteins and the AQP0 COOH-terminal peptide. Filensin (94 kDa) is processed into 50 and 38 kDa proteins in normal lens. Because the degraded part is located in the tail domain, the anti-filensin outer tail domain antibody only reacts with 94 kDa filensin in lens extract and does not react with the 50 or 38 kDa proteins (Figure 1A; lane 1). Filensin (94 kDa) interacted with the recombinant AQP0 COOH-terminal peptide (Figure 1A; lane 2). Anti-filensin antibody did not interact with recombinant AQP0 in the absence of lens extract (Figure 1A; lane 3).

The anti-filensin rod antibody used to investigate the interaction between lens extract proteins and the AQP0 COOH-terminal peptide revealed three different bands for filensin at 94, 50 and 38 kDa in the urea fraction of rat lens extract (Figure 1B; lane 1). We have shown previously that the 94 kDa protein is processed into two smaller fragments of 50 and 38 kDa, which both contain the rod domain, while the 50 kDa fragment also contains a portion of the tail domain adjacent to the rod domain [22]. In the presence of lens extract, the 94 and 50 kDa filensin bands interacted with purified AQP0 COOH-terminal peptide but the 38 kDa filensin fragment did not (Figure 1B; lane 2). In the absence of lens extract, purified GST-AQP0-C did not react with the antibody (Figure 1B; lane 3). These results indicated that the AQP0 COOH-terminal peptide interacts with the tail region of filensin since the 94 and 50 kDa filensin species contain both the rod and tail regions, while the 38 kDa filensin does not include the tail.

Using the GST-AQP0-C construct as a template, three plasmids for pseudophosphorylated forms of the COOH-terminal peptide, 1S/E-AQP0, 3S/E-AQP0, and 5S/E-AQP0 (one, three and five pseudophosphorylated sites, respectively), were constructed, expressed in *E. coli* and purified with glutathione beads before incubation with lens extract. Western blotting with an anti-filensin antibody was used to analyze the interaction between filensin and the pseudophosphorylated AQP0-COOH-terminal peptides. None of the pseudophosphorylated AQP0-COOH-terminal peptides reacted with the anti-filensin antibody in the absence of lens extract (Figure 1B; lanes 5, 7, and 9). In the presence of lens extract, both the 94 kDa and 50 kDa filensin fragments interacted with 1S/E-AQP0, 3S/E-AQP0, and 5S/E-AQP0 but did not interact with the 38 kDa filensin fragment (Figure 1B; lanes 4, 6, and 8, respectively). No differences in the interaction with filensin were detected between the AQP0 COOH-terminal peptide and its pseudophosphorylated proteins. As a further control, GST expressed from pGEX6P-1 without the AQP0 fusion protein did not interact with the filensin in the lens extract (Figure 1B; lane 10).

### Interaction between recombinant filensin and AQP0 in lens extract

A GST-filensin tail domain fusion construct (GST-filensin-tail) and a GST-filensin rod domain fusion construct (GST-filensin-rod) were expressed in *E. coli* and their interactions with the AQP0 COOH-terminal peptide were analyzed by western blotting using an anti-AQP0 antibody.

**Figure 2:**
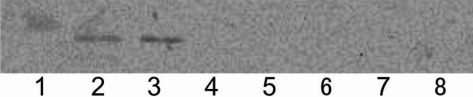
Interaction between GST-filensin-tail or GST-filensin-rod and AQP0 in rat lens extract. The interaction between purified recombinant GST-filensin-tail or GST-filensin-rod with lens AQP0 was detected by western blotting with an anti-AQP0 COOH-terminal antibody. Lane 2 shows a 23 kDa AQP0 protein band present in the Triton-X100 extract fraction from rat lens. Incubation of GST-filensin-tail with the Triton-X100 extract fraction from rat lens revealed an AQP0 protein band at 23 kDa, illustrating the interaction between the filensin tail region and AQP0 (lane 3). In the absence of lens extract, the purified GST-filensin-tail construct did not react with the anti-AQP0 antibody (lane 4). There was no interaction between the purified filensin rod domain and AQP0 in the lens extract fraction (lane 5). In the absence of lens extract, GST-filensin-rod conjugated to glutathione beads did not contain a peptide that reacted with the anti-AQP0 antibody (lane 6). No AQP0 band was detected when GST alone was incubated with lens extract (lane 7). Anti-AQP0 antibody did not react with GST (lane8). Lane 1 shows molecular weight markers.

AQP0, a protein of lens extract, interacted with the GST-filensin-tail construct to reveal a band at 23 kDa (Figure 2; lane 3). In the absence of lens extract, the GST-filensin-tail construct did not react with the anti-AQP0 antibody (Figure 2; lane 4), and in rat lens extract alone, the anti-AQP0 antibody detected a protein band at 23 kDa (Figure 2; lane 2). The GST-filensin-rod construct did not interact with AQP0 present in rat lens extract (Figure 2; lane 5). In the absence of lens extract, the GST-filensin-rod construct showed no interaction with the AQP0 antibody (Figure 2; lane 6). Taken together, these results suggest that the filensin tail region interacts with AQP0 but not with the filensin rod domain. Also, GST alone did not interact with lens AQP0 (Figure 2; lane 7). Anti-AQP0 antibody did not react with GST (Figure 2; lane8).

### Effect of pH on the interaction between AQP0 and the filensin tail domain

To determine whether AQP0 and filensin interact directly with one another, western blotting analysis with an anti-filensin tail antibody was used to study the interaction between the recombinant filensin tail region and the AQP0 COOH-terminal peptide. To investigate the effect of pH on this interaction, GST-AQP0-C was incubated with GST-filensin-tail in different pH buffers: pH 7.0, 7.5, 8.0, or 8.5.

**Figure 3:**
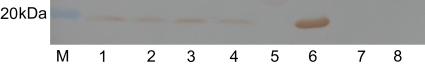
Effect of pH on the interaction between recombinant GST-AQP0-C and the recombinant filensin tail peptide. The filensin tail peptide was cleaved from the purified recombinant GST-fusion construct using preScission protease. Recombinant GST-AQP0-C and the recombinant filensin tail peptide were then incubated in different pH buffers and the interaction between the AQP0 COOH-terminal peptide and the filensin tail peptide was detected by western blotting with an anti-filensin tail antibody. Lanes 1, 2, 3, and 4 show the interaction between the filensin tail peptide and GST-AQP0-C in buffers with pH 7.0, 7.5, 8.0, and 8.5, respectively. Lane 5, GST alone incubated with the recombinant filensin tail peptide. Lane 6, purified filensin tail peptide cleaved from the GST-filensin tail fusion construct. Lane 7, GST-AQP0-C alone. Lane 8, GST alone.

After purification of the GST-filensin-tail construct with glutathione beads, the filensin tail domain was collected in the supernatant following cleavage from GST using preScission protease (Figure 3; lane 6). GST-AQP0-C, which was constructed using pGEX 5x-1, was expressed in *E. coli* and purified by glutathione beads, and did not interact with the anti-filensin tail antibody (Figure 3; lane 7). The purified filensin tail peptide was then added to glutathione beads conjugated to the GST-AQP0-C and dialyzed against buffers of pH 7.0, 7.5, 8.0, or 8.5 (Figure 3; lanes 1, 2, 3, and 4, respectively). The interactions were detected by western blotting using the anti-filensin tail antibody and no differences were observed between the four pH buffers. GST alone did not interact with the filensin tail peptide (Figure 3; lane 5) or with the anti-filensin tail antibody (Figure 3; lane 8).

### Effect of filensin and AQP0 interaction on water permeability

**Figure 4:**
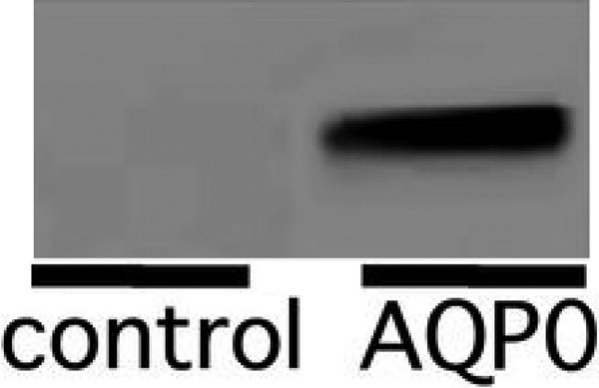
*Xenopus* oocyte expressing AQP0 AQP0 in *Xenopus* oocytes was injected with cRNA AQP0 was detected by western blotting.

**Figure 5:**
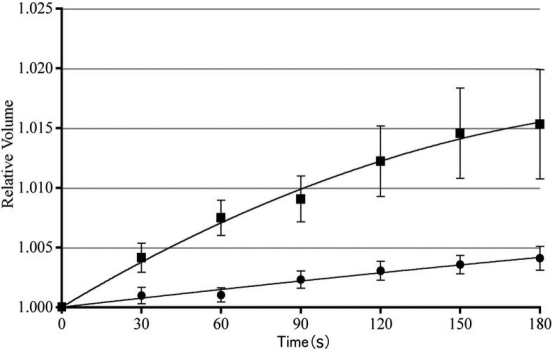
Change of diameter of *Xenopus* oocytes expressing AQP0 *Xenopus* oocytes expressing AQP0 were immersed in hypotonic solution (30% ND96 soluion). The diameter of *Xenopus* oocytes injected with 25 ng of AQP0 cRNA (closed square) or water (closed circle) was measured. The oocytes expressing AQP0 permeated more water than control.

**Figure 6:**
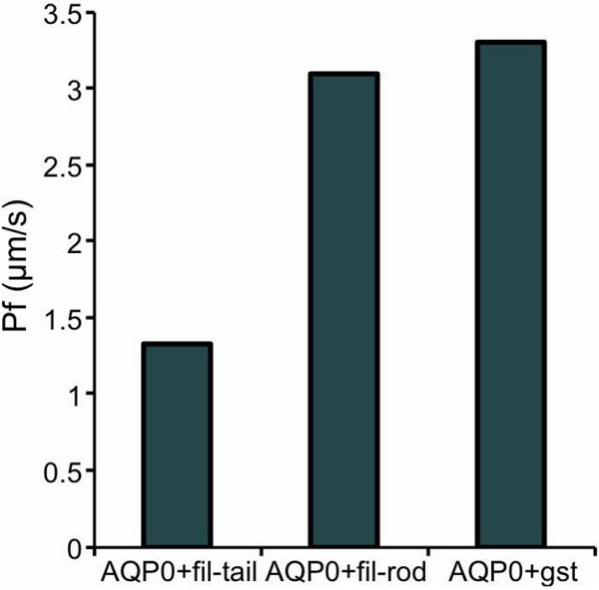
Water permeability of AQP0 expressed in *Xenopus* oocytes. The osmotic water permeability (Pf) of AQP0 expressed on *Xenopus* oocyte membranes was determined by measuring the change in oocyte diameter. To balance the loading of ribosomes and obtain the same amount of transcribed AQP0 in each reaction, 12.5 ng cRNA of GST was co-injected as a blank with 12.5 ng cRNA of AQP0 (AQP0+GST). The water permeability was reduced when *Xenopus* oocytes were co-injected with 12.5 ng filensin COOH-terminal peptide cRNA and 12.5 ng of AQP0 cRNA, however, there was no significant difference between the water permeability value for AQP0+GST and the value obtained when 12.5 ng filensin rod domain cRNA was co-injected with 12.5 ng AQP0 (AQP0+fil rod) cRNA. These results suggested that the filensin tail region interacted with AQP0 to reduce the water permeability of AQP0, while the filensin rod peptide had no effect.

*Xenopus* oocytes were expressed AQP0 on its membrane by injectng with cRNA of AQP0. Western blotting analysis with anti-AQP0 antibody was used to confirm the expression of AQP0 on *Xenopus* oocytes (Figure 4). The *Xenopus* oocytes expressing AQP0 were transferred to 30% ND96 solution. After transfer to the hypotonic solution, the oocyte swelling was photographed and the volume of each oocyte was calculated by measuring its diameter. The diameter of *Xenopus* oocytes injected with AQP0 cRNA was greater than that of oocytes injected with water (Figure 5). The amount of protein transcribed from the cRNA should be proportional to the amount of injected cRNA; however, it is possible that the amount of transcribed protein could be limited by the transcriptional ability of the ribosome. Therefore, to ensure that the ribosomes were loaded equally and to obtain the same amount of transcribed AQP0, 12.5 ng of GST cRNA was co-injected as a blank with 12.5 ng of AQP0 cRNA (AQP0+GST). GST did not interact with AQP0 (Figure 2; lane 7). The water permeability of oocytes injected with AQP0+GST cRNA was calculated using the water permeability of oocytes injected GST cRNA as a reference. The water permeability was reduced when a cRNA for the filensin tail peptide was co-injected with AQP0 into *Xenopus* oocytes (AQP0+fil tail) but did not change significantly when the filensin rod peptide cRNA was co-injected with AQP0 (AQP0+fil rod; Figure 6). The data were also calculated using the water permeability of oocytes injected with filensin-tail cRNA or filensin-rod cRNA. The water permeability of oocytes injected with filensin-tail cRNA or filensin-rod cRNA was used to as a reference to calculate the water permeability of oocytes injected with AQP0+fil tail cRNA or AQP0+fil rod cRNA. These results suggested that the filensin rod domain did not interact with AQP0 and that the filensin tail peptide interacted with AQP0 to reduce AQP0 permeability.

## Discussion

### Interaction between filensin and AQP0

This study examined the interaction between the lens membrane protein, AQP0, and the cytoskeletal protein, filensin. Although the interaction between AQP0 and filensin has already been demonstrated [29], this investigation shows that the COOH-terminal region of AQP0 interacts directly with the tail region of filensin. Protein interactions in vitro do not always reflect the actual interactions that occur in vivo since they can be influenced by the ionic strength and/or pH of the buffer used in vitro. There is also a possibility that the recombinant AQP0 peptide may interact with many proteins nonspecifically. Furthermore, the interaction between AQP0 and filensin might be indirect with AQP0 interacting with an unknown protein that then interacts with filensin.

This study has shown that GST-AQP0-C interacted with lens filensin and that GST-filensin-tail interacted with lens AQP0. In addition, GST-AQP0-C also interacted with the filensin tail peptide purified from the GST-filensin-tail construct. These results strongly suggested that the COOH-terminal region of AQP0 interacts with the filensin COOH-terminal tail region and that the interaction is direct. GST alone did not interact with lens proteins and GST-AQP0-C did not interact with the filensin rod domain indicating that the AQP0 COOH-terminal peptide reacted specifically with the filensin tail peptide rather than non-specifically with lens proteins.

Filensin is a 94 kDa intermediate filament protein and is processed into two smaller molecular weight proteins of 50 and 38 kDa in the normal lens [22,34]. In the normal rat lens, the filensin rod domain is localized to the membrane-lining region in shallow cortex cells and to the central region of the cytoplasm in deep cortex cells. In cells of the deep cortex, the filensin tail domain is localized to subcellular membranes, which suggests that it is the filensin tail domain that binds to AQP0 at the lens plasma membrane, rather than the rod region.

### Protein interactions with AQP0

There have been several reports of interactions between AQP0 and various lens proteins. Calmodulin has been shown to interact with the COOH-terminal region of AQP0 [10,14,35], and AQP0 also binds alpha crystallin [36,37], filensin and CP49 [22]. Calmodulin interacts with AQP0 to reduce water permeability and the phosphorylation of AQP0 at specific serine residues in the COOH-terminal peptide is known to lower its binding affinity with calmodulin [10,11,13,14]. These serine residues show 10 to 15% phosphorylation in vivo in the normal lens cortex [38]. Thus, most of AQP0 is considered active for the binding of calmodulin. Calmodulin dissociates from AQP0 when the AQP0 COOH-terminal peptide is phosphorylated and the water permeability of AQP0 increases in the absence of calmodulin. This phosphorylation and dephosphorylation may help to regulate the water permeability and the amount of water in the lens. AQP0 is known to interact with calmodulin, but whether the AQP0 COOH-terminal region binds to both calmodulin and filensin simultaneously is unknown.

### Regulation of AQP0 water permeability

This study has shown that filensin interacts with AQP0 and that this interaction reduces the water permeability of AQP0. The water permeability of AQP0 is very low compared to other members of the aquaporin family, such as AQP1 [8]. However, since the lens is surrounded by a capsule and lens epithelial cells continue to divide and differentiate, the turnover of most molecules in lens cells is likely to be slow. In addition, in lens fiber cells, AQP0 accounts for more than 60% of the membrane proteins. Thus, a protein such as AQP0, even with its low water permeability, would play a significant role in regulating the water content of the lens since it is present in large quantities. In addition, the binding of filensin and/or calmodulin reduces the water permeability further, and it is suggested that, as a low activity cell, lens fiber cells do not require rapid water transport.

This study demonstrated that the binding between AQP0 and filensin was not affected by the pseudophosphorylation of the AQP0 COOH-terminal peptide, suggesting that the binding between AQP0 and filensin is stable and strong. Both AQP0 and filensin are expressed at the start of lens fiber cell differentiation and they may play important roles in the elongation of lens fiber cells and/or in the placement of the beaded filament and AQP0 in lens fiber cells.

There have been reports that the water permeability (Pf) of AQP0 is regulated by pH and Zn^2+^, Ni^2+^, and Ca^2+^ ions [9,11,39,40]. Regulation by pH has been linked to histidine residues on the extracellular loops of AQP0 [9,41] and it has been shown that decreasing the pH from 7.5 to 6.5 increases the permeability of AQP0-injected oocytes [9]. This study examined the effect of pH on the interaction between AQP0 and filensin but found that pH (from pH 6.5 to pH 8.5) had no effect on the AQP0/filensin interaction. Histidine 40 in the AQP0 A loop is required for the pH sensitivity of AQP0 water permeability [9], and although the AQP0 COOH-terminal peptide used in the rat GST-AQP0-C construct did not contain histidine, it did contain 12 amino acids that are acidic and anodic. These amino acids change their electric charge depending on the pH of the solution. The fact that, in this study, changes in pH had no effect on the AQP0/filensin interaction also suggested that the binding between AQP0 and filensin is stable and strong.

This study has shown that the water permeability of AQP0 is regulated by filensin, in addition to the roles played by calcium and calmodulin. The lens does not contain blood vessels and all its nutrition is achieved through cell-cell communication, which is why lens fiber cell membranes contain many channels and junction proteins compared with other cell types. In addition, since lens fiber cells have a very low metabolism and persist throughout the life of the organism, it is likely that water permeability needs to be very slow and well regulated. AQP0 constitutes more than 60% of the total membrane protein content of fiber cells [5,6] and AQP0 permeates water very slowly compared to other aquaporins [2,8]. The AQP0/filensin-tail interaction reduces water permeability even more. Very slow water permeation may help regulate lens water turnover. The precise regulation of water permeability is probably important for lens physiology and transparency.
